# Neuroprotective Effects of Adipose-Derived Stem Cells Are Maintained for 3 Weeks against Ischemic Damage in the Rabbit Spinal Cord

**DOI:** 10.1155/2014/539051

**Published:** 2014-01-29

**Authors:** Seung Myung Moon, Woosuk Kim, Jin Young Chung, Wooseok Im, Dae Young Yoo, Hyo Young Jung, Moo-Ho Won, Jung Hoon Choi, In Koo Hwang

**Affiliations:** ^1^Department of Neurosurgery, Dongtan Sacred Heart Hospital, College of Medicine, Hallym University, Hwaseong 445-170, Republic of Korea; ^2^Department of Anatomy and Cell Biology, College of Veterinary Medicine and Research Institute for Veterinary Science, Seoul National University, Seoul 151-742, Republic of Korea; ^3^Department of Veterinary Internal Medicine and Geriatrics, College of Veterinary Medicine, Kangwon National University, Chuncheon 200-701, Republic of Korea; ^4^Department of Neurology, Seoul National University Hospital, Seoul 110-744, Republic of Korea; ^5^Department of Neurobiology, School of Medicine, Kangwon National University, Chuncheon 200-701, Republic of Korea; ^6^Department of Anatomy, College of Veterinary Medicine, Kangwon National University, Chuncheon 200-701, Republic of Korea

## Abstract

In the previous study, we demonstrated that adipose-derived stem cells (ASCs) have neuroprotective effects against ischemic damage in the ventral horn of L_5-6_ levels at 3 days after ischemia/reperfusion. In the present study, we expanded our observations for 3 weeks after ischemia/reperfusion to rule out the possibility of delayed neuronal death in several days after ischemia/reperfusion. Transient spinal cord ischemia was induced by a 15 min aortic artery occlusion in the subrenal region and rabbit ASCs were administered intrathecally into recipient rabbits (2 × 10^5^) immediately after reperfusion. Transplantation of ASCs improved the neurological motor functions of the hindlimb 3 weeks after ischemia/reperfusion. Similarly, the cresyl violet-positive neurons were significantly increased at 3 weeks after ischemia/reperfusion compared to that in the vehicle (artificial cerebrospinal fluid)-treated group. The transplantation of ASCs significantly reduced reactive microglia induced by ischemia at 3 weeks after ischemia/reperfusion. In addition, transplantation of ASCs maintained the brain-derived neurotrophic factor (BDNF) levels 3 weeks after ischemia/reperfusion. These results suggest that the neuroprotective effects of ASCs are maintained 3 weeks after ischemia/reperfusion by modulating microgliosis and BDNF levels in the spinal cord.

## 1. Introduction

Paraplegia, one of the serious complications in thoracoabdominal aorta aneurysms, is induced by transient spinal cord ischemia. It affects approximately 12 in 100,000 people in the general population, but the prognosis is very poor [1]. Transient spinal cord ischemia induces the excitatory amino acids and calcium influx into the neurons and finally causes neuronal death in the ventral horn of spinal cord. In addition, neurotrophic factors transiently increased after spinal cord ischemia, but they significantly decreased in the damaged region several days after ischemia/reperfusion and this led to decrease of regenerative processes [2–4].

Spinal cord ischemia has a low therapeutic efficacy and the standard therapy in clinical trial is aspirin [5]. It has been shown that some anti-inflammatory agents had therapeutic effects in animal studies. Recently, stem cell transplantation has been highlighted because stem cells can contribute to tissue regeneration, restructuring of neuronal networks, and enhancement of endogenous repair [6–9]. Stem cells are broadly classified into 2 types: embryonic stem cell from inner cell mass of blastocysts and adult stem cells from various tissues including bone marrow and adipose tissue. Among these stem cells, adipose-derived stem cells (ASCs) can be easily obtained from liposuction waste tissue in plastic surgery. ASCs have shorter doubling times than bone marrow derived stem cells [10].

Protective effects of ASCs transplantation against ischemic damage were reported in the brain [11, 12]. In addition, free media of ASCs also have neuroprotective effects against ischemic damage in the brain [13]. In a previous study, we demonstrated that ASCs have neuroprotective effects in the spinal cord within 3 days after ischemia/reperfusion [4]. However, there were no studies on the tracking of the neuroprotective effects on spinal cord within several weeks after ischemia/reperfusion. In this study, therefore, we investigated the effects of ASCs on neuronal damage within 3 weeks after ischemia/reperfusion in the rabbit spinal cord.

## 2. Materials and Methods

### 2.1. Animals

Twenty-seven male New Zealand white rabbits (1.5–2.0 kg) were obtained from the Experimental Animal Center (Cheonan Yonam College, Cheonan, South Korea). They were housed in a conventional state under adequate temperature (21°C) and humidity (60%) controls with a 12 h light/12 h dark cycle and could freely access food and tap water. The handling and care of the animals conformed to guidelines established to comply with current international laws and policies (NIH Guide for the Care and Use of Laboratory Animals, NIH Publication Number 85-23, 1985, revised 1996) and were approved by the Institutional Animal Care and Use Committee (IACUC) of Kangwon National University in Chuncheon, South Korea. All experiments were conducted with an effort to minimize the number of animals used and the suffering caused by the procedures used in the present study.

### 2.2. Cell Preparation

ASCs were isolated and cultured as mentioned in a previous study and we also confirmed the characteristics of stem cells in a previous study by flow cytometry analysis and differentiation into adipocyte [4].

### 2.3. Induction of Transient Spinal Cord Ischemia

The animals were anesthetized with a mixture of 2.5% isoflurane (Baxtor) in 33% oxygen and 67% nitrous oxide. A ventral midline incision was made in the abdomen. The abdominal aorta was isolated underneath the left renal artery and occluded using a nontraumatic aneurysm clip to conduct transient spinal cord ischemia according to a modified method reported by Kiyoshima et al. [14]. After 15 min of occlusion, the aneurysm clip was removed, and the restoration of blood flow (reperfusion) was observed from the abdominal aorta. Body temperature was maintained under free-regulating or normothermic (38.7 ± 0.3°C) conditions, monitored with a rectal temperature probe (TR-100; Fine Science Tools, Foster City, CA), and using a thermometric blanket before, during, and after the surgery until the animals completely recovered from anesthesia. Thereafter, the animals were kept on the thermal incubator (Mirae Medical Industry, Seoul, South Korea) to maintain body temperature until the animals were euthanized. Sham-operated animals were subjected to the same surgical procedures except for the abdominal aorta occlusion. All animals received the insertion of catheter because ischemia damaged the micturition reflex.

### 2.4. Cell Transplantation

Rabbits were divided into 3 groups (*n* = 9 in each group): (1) sham-ischemia (sham) group as a control group, (2) vehicle (artificial cerebrospinal fluid)-treated (vehicle) ischemia group, and (3) ASCs-treated (ASCs) group. The animals received a single injection of ASCs immediately after reperfusion and the animals were sacrificed at 3 weeks after ischemia/reperfusion. The ASCs (2 × 10^5^) were suspended in 5 *μ*L phosphate-buffered saline (PBS), and they were then transplanted intrathecally into the cisterna magna. The same volume of vehicle was injected in the same way of ASCs treatment. The technique of intrathecal injection was used as previously reported [15, 16]. There were no obvious behavioral sequelae such as locomotor, feeding, or drinking after intrathecal injection of ASCs.

### 2.5. Neurological Function Assessment

For assessment of neurological function, modified Tarlov criteria were used: 0, no voluntary hindlimb function; 1, only perceptible joint movement; 2, active movement but unable to stand; 3, able to stand but unable to walk; or 4, complete normal hind-limb motor function [17, 18]. All assessments were performed to ensure objectivity in blind conditions by two observers for each experiment, carrying out assessments in the sham, vehicle-ischemia, and ASCs-ischemia groups under the same conditions. Neurological functions were assessed at 1, 2, and 3 weeks after reperfusion because the progressive neuronal death may occurr within these weeks.

### 2.6. Cresyl Violet Staining

A histological evaluation of the spinal cord was performed at 3 weeks after ischemia/reperfusion using cresyl violet staining method to stain the surviving neurons in the spinal cord. After the last neurological assessment, the sham, vehicle-ischemia, and ASCs-ischemia groups were randomly divided into 2 groups, histological (*n* = 5 in each group) and biochemical groups (*n* = 4 in each group). The animals in histological groups were anesthetized with 1 g/kg urethane (Sigma, St. Louis, MO) and perfused transcardially with 0.1 M phosphate buffered saline (PBS, pH 7.4) followed by 4% paraformaldehyde in 0.1 M phosphate-buffer (PB, pH 7.4). The animals' spinal cords were removed, and the 5-6th lumbar segments (L_5-6_) of the spinal cord were cryoprotected by infiltration with 30% sucrose overnight and 30 *μ*m thick spinal cord sections in the coronal plane were serially cut using a cryostat (Leica, Wetzlar, Germany). The sections were stained with cresyl violet acetate as previously described [19].

The measurement of overall size of spinal cord and the number of cresyl violet positive cells in all the groups was performed using an image analyzing system equipped with a computer-based CCD camera (software: Optimas 6.5, CyberMetrics, Scottsdale, AZ).

### 2.7. Immunohistochemistry for CD11b

The sections were sequentially treated with 0.3% hydrogen peroxide (H_2_O_2_) in PBS and 10% normal goat serum in 0.1 M PBS. They were next incubated with diluted mouse anti-CD11b (1 : 100, Chemicon International, Temecula, CA) overnight and subsequently exposed to biotinylated goat anti-mouse IgG (diluted to 1 : 200, Vector, Burlingame, CA) and streptavidin peroxidase complex (diluted to 1 : 200, Vector). Then, the sections were visualized by reaction with 3,3′-diaminobenzidine tetrahydrochloride (Sigma) in 0.1 M Tris-HCl buffer (pH 7.2) and mounted on gelatin-coated slides. The sections were mounted in Canada Balsam (Kanto, Tokyo, Japan) after dehydration.

### 2.8. Measurement of Brain-Derived Neurotrophic Factor (BDNF) Level

To elucidate the effects of ASCs on BDNF levels in the sham, vehicle-ischemia, and ASCs-ischemia groups (*n* = 4 in each group), BDNF levels were measured using commercial kits purchased from Uscn Life Science Inc. (Wuhan, China) at 3 weeks after ischemia/reperfusion. All spectrophotometric readings were performed using a spectrophotometer (DU 640B, Beckman, Fullerton, CA). All assays were conducted in triplicate.

### 2.9. Statistical Analysis

The data presented represent the means of the experiments performed for each experimental investigation. The differences among the means were statistically analyzed by a one-way analysis of variance followed by a Tukey's multiple range method to elucidate differences among groups. Statistical significance was considered at *P* < 0.05.

## 3. Results

### 3.1. Neurological Outcomes

**Figure 1:**
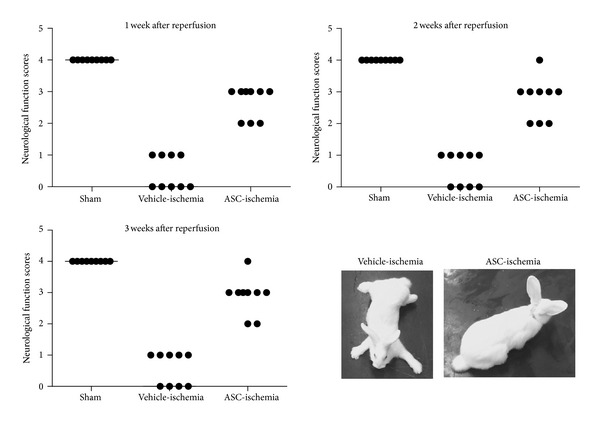
Neurological function scores by modified Tarlov criteria in the sham, vehicle-ischemia, and ASC-ischemia groups at 1, 2, and 3 weeks after ischemia/reperfusion (*n* = 5 per group). In addition, the representative pictures of posture in rabbit of vehicle-ischemia and ASC-ischemia groups at 3 weeks after ischemia/reperfusion.

In this study, we observed hind-limb motor function scores at 1, 2, and 3 weeks after ischemia/reperfusion (Figure 1). In the sham group, all animals showed the score of 4 and there were no significant changes in posture and gait during experiments. In the vehicle-ischemia group, most of the animals showed significant malfunctions in posture and function of hindlimbs from 24 h after ischemia/reperfusion. At 1, 2, and 3 weeks after ischemia/reperfusion, most of the animals showed complete paraplegia in both hind-limbs, and the mean neurological score in this group was 0.44–0.56 in these time points after ischemia/reperfusion. In the ASC-ischemia group, most animals could stand although some animals showed extension and enhanced muscle tone in hind-limb. In this group, the mean neurological scores were 2.67–2.89, which were much improved compared to those in the vehicle-ischemia group at all times after ischemia/reperfusion (Figure 1).

### 3.2. Neuroprotective Effect

**Figure 2:**
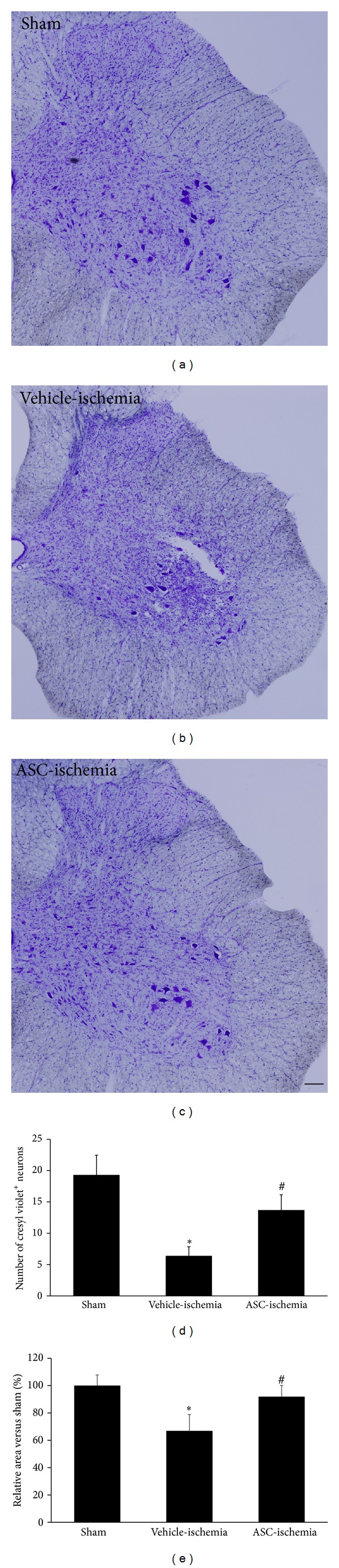
Cresyl violet staining in the ventral horn of the L_5_-L_6_ spinal cord in the sham (a), vehicle-ischemia (b), and ASC-ischemia (c) groups at 3 weeks after ischemia/reperfusion. In the sham group, cresyl violet positive neurons are thoroughly detected in the spinal cord. In the vehicle-ischemia group, cresyl violet positive neurons are significantly decreased in the ventral horn of spinal cord. In addition, the size of spinal cord is small compared to that in the sham group. In the ASC-ischemia group, cresyl violet-positive neurons are abundant in the ventral horn. Scale bar = 250 *μ*m. The mean number of cresyl violet-positive neurons (d) and the overall size of spinal cord (e) per section of each group (*n* = 5 per group; *P < 0.05 versus the sham group; ^#^ *P* < 0.05 versus the vehicle-ischemia group). All data are shown as the means ± standard errors of means (SEM).

In the sham group, many cresyl violet positive neurons were detected throughout the gray matter of spinal cord at 3 weeks after the sham operation (Figure 2(a)). In this group, the mean number of cresyl violet-positive neurons was 19.3 per section in the ventral horn of the spinal cord. In the vehicle-ischemia group, the overall size of spinal cord was significantly decreased compared to that in the sham group (Figure 2(e)). In addition, the number of cresyl violet positive neurons in the ventral horn of spinal cord was significantly decreased compared to that in the sham group and was 6.4 per section (Figures 2(b) and 2(d)). In the ASC-ischemia group, the overall size of spinal cord was slightly decreased compared to that in the sham group and the number of cresyl violet positive neurons in the ventral horn was abundant compared to that in the vehicle-ischemia group (Figures 2(d) and 2(e)). In this group, the mean number of cresyl violet-positive neurons was 13.7 per section in the ventral horn (Figures 2(c) and 2(d)).

### 3.3. Reactive Microgliosis

**Figure 3:**
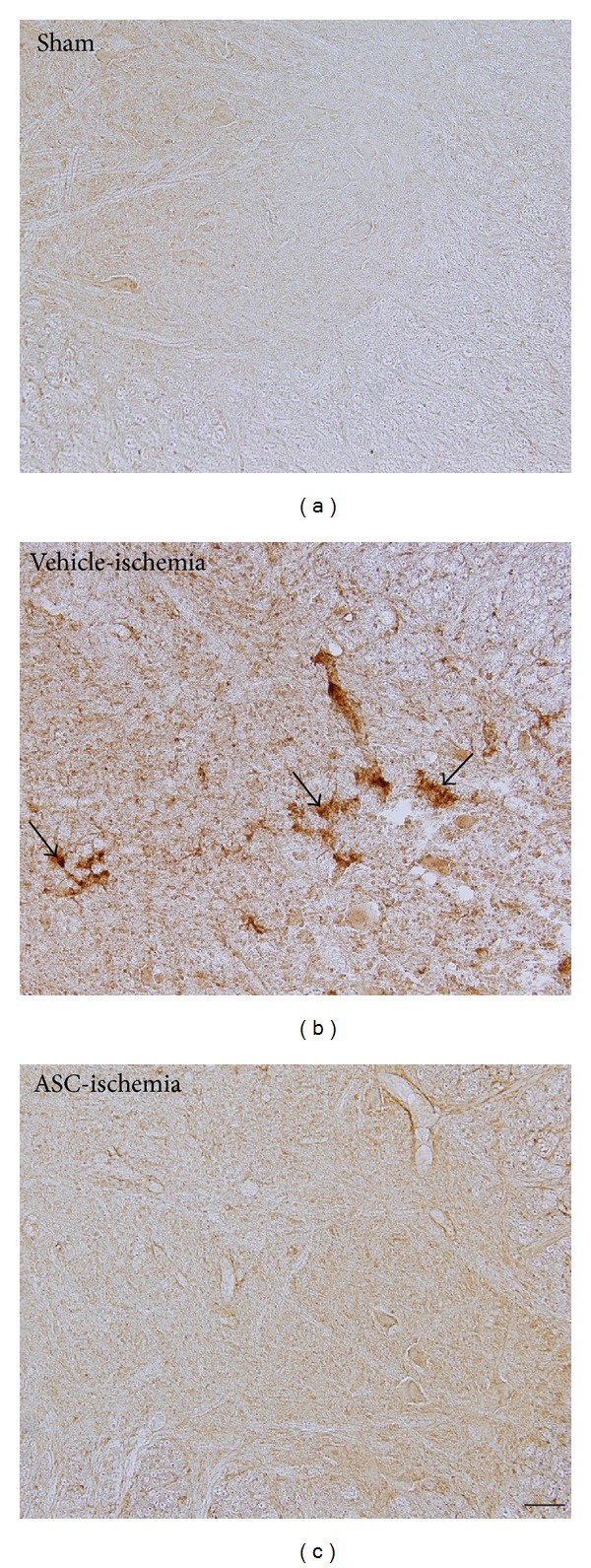
Immunohistochemical staining for CD11b in the ventral horn of the L_5_-L_6_ spinal cord in the sham (a), vehicle-ischemia (b), and ASC-ischemia (c) groups at 3 weeks after ischemia/reperfusion. In the sham group, CD11b-immunoreactive microglia are hardly detected. In the vehicle-ischemia group, massive CD11-immunoreactive microglia are shown (arrows). In the ASC-ischemia group, CD11b-immunoreactive microglia are not detected. Scale bar = 100 *μ*m.

In the sham group, CD11b immunoreactive structure was not observed in the ventral horn of the spinal cord at 3 weeks after the sham operation (Figure 3(a)). In the vehicle-ischemia group, CD11b immunoreactive structures were prominently detected in the ventral horn of spinal cord and these CD11b immunoreactive structures were microglia based on morphology (Figure 3(b)). In the ASC-ischemia group, some CD11b immunoreactive microglia were observed; however, its immunoreactivity significantly decreased compared to that in the vehicle-ischemia group and nearly disappeared in the ventral horn of spinal cord (Figure 3(c)).

### 3.4. BDNF Levels

**Figure 4:**
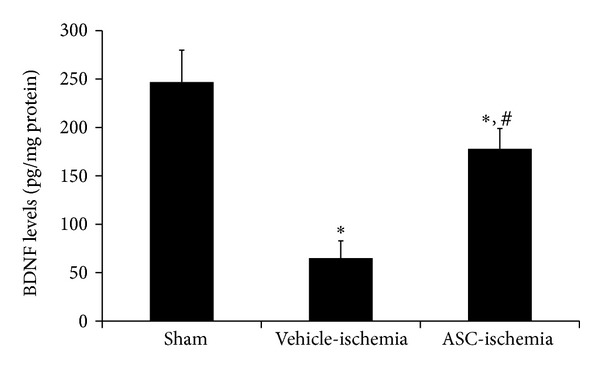
Levels of BDNF in the L_5_-L_6_ spinal cord in the sham, vehicle-ischemia, and ASC-ischemia groups at 3 weeks after ischemia/reperfusion (*n* = 4 per group; *P < 0.05 versus the sham group; ^#^ *P* < 0.05 versus the vehicle-ischemia group). All data are shown as the means ± SEM.

In the sham group, the BDNF level from L_5_-L_6_ spinal cord homogenates was 247 pg/mg protein. In the vehicle-ischemia group, the BDNF level was significantly decreased compared to the sham group and the BDNF level from L_5_-L_6_ spinal cord homogenates was 65 pg/mg protein. In the ASC-ischemia group, the BDNF level was significantly increased compared to the vehicle-ischemia group. In this group, the BDNF level from L_5_-L_6_ spinal cord homogenates was 178 pg/mg protein (Figure 4).

## 4. Discussion

In previous study, we isolated the rabbit ASC from fat sample in thigh region, and subcutaneous tissue and detached ASCs at passage 3 showed surface marker profiles of ASCs. In addition, this ASC could differentiate into adipocytes in adipogenesis differentiation medium. The administration of ASCs significantly reduces the neurological scores and morphological findings in the spinal cord at 72 h after ischemia/reperfusion [4]. In the present study, we investigated the long-term effects of ASCs against spinal cord ischemia at 3 weeks after ischemia/reperfusion to rule out the possibility of delayed neuronal death in ASC-ischemia groups. Animals which received ASCs via intrathecal route show significant differences in behavior compared to that in the vehicle-treated group. The urine should be squeezed out in the vehicle-ischemia group because of damage in micturition reflex. However, ASC-ischemia group demonstrated normal micturition reflex and could stand at 1–3 weeks after ischemia/reperfusion.

At 3 weeks after ischemia/reperfusion, cresyl violet-positive neurons were significantly decreased in the ventral horn of spinal cord. In addition, the overall size of spinal cord was significantly decreased compared to that in the sham group. This result suggests that transient spinal cord ischemia reduces the cell number as well as the spinal cord volume. It has been reported that the hippocampal CA1 region was severely shrunken due to the absence of pyramidal cells 1 year after transient global ischemia by transient 4-vessel occlusion in rats [20]. In the present study, significant increase of the neurological scores and preserved cresyl violet-positive neurons after transplantation of homologous ASCs were observed. In addition, the overall size of spinal cord was similar to that in the sham group. This result suggests that the homologous ASCs have neuroprotective effects and the effect persisted up to 3 weeks after ischemia/reperfusion. It has been reported that ASC cluster or human embryonic stem cell line-derived neural precursors improved spinal cord injury [21]. Human embryonic stem cell line-derived neural precursors can differentiate into neuroblasts at 2–4 weeks after grafting and into mature neurons at 4–8 weeks after grafting in the ischemia-injured spinal cord in rats or in naive immunosuppressed minipigs [22]. However, one of grafting stem cells consistently leads to teratoma formation at 2–6 weeks after cell transplantation.

Transient forebrain or spinal cord ischemia induces neuronal damage by various mechanisms including the inflammation to brain or spinal cord. Transient spinal cord ischemia induces the activation of microglia [23]. In this study, we also observed reactive microgliosis in the spinal cord 3 weeks after ischemia/reperfusion. CD11b immunoreactive microglia were prominently observed in the ventral horn of spinal cord, and the administration of ASCs significantly reduced the CD11b immunoreactive microglia. This result suggests that ASCs may reduce the activation of microglia in the ventral horn of spinal cord induced by cell death after transient spinal cord ischemia.

It has been reported that mesenchymal stem cells secrete various trophic factors that facilitate neuroprotection, angiogenesis, or neurogenesis [10, 24–28]. Among these trophic factors, BDNF showed a beneficial effect on the survival and maintenance of neuronal functions in both the peripheral and the central nervous system [29–31]. In the present study, the BDNF levels were higher in the ventral horn of spinal cord in ASC-ischemia group than those in the vehicle-ischemia group. This result suggests that the increased levels of BDNF were possibly associated with the functional improvement by ASC intrathecal transplantation into the spinal cord in the rabbits. This result was supported by previous study that engrafted neural stem cells expressed BDNF and increased the level of BDNF mRNA in injured site following transplantation [32].

ASCs have neuroprotective effects against spinal cord ischemia and these effects persisted up to 3 weeks after ischemia/reperfusion by reducing the microglial activation and increasing BDNF levels.
